# Cytotoxic T Cell Responses Induced by CS1/CRT Fusion DNA Vaccine in a Human Plasmacytoma Model

**DOI:** 10.3389/fonc.2020.587237

**Published:** 2020-11-20

**Authors:** Xueshi Ye, Wanli Li, Jinwen Huang, Lifei Zhang, Ye Zhang

**Affiliations:** ^1^ Department of Hematology, Sir Run Run Shaw Hospital, School of Medicine, Zhejiang University, Hangzhou, China; ^2^ Department of Orthopedics, The Second Affiliated Hospital, School of Medicine, Zhejiang University, Hangzhou, China

**Keywords:** myeloma, immunotherapy, DNA vaccine, CS1, calreticulin

## Abstract

To date, multiple myeloma remains an incurable disease. Immunotherapy is an encouraging option in the development of multiple myeloma (MM) therapy. CS1 is a specific myeloma antigen, which is highly expressed in myeloma cells. Calreticulin (CRT) is a key determinant of cell death, which can influence antigen presentation and promote cellular phagocytic uptake. In the current study, we constructed a DNA vaccine encoding both CS1 and CRT. Our results show that the PcDNA3.1-CS1/CRT vaccine was able to induce cytotoxic T cell responses against myeloma cells *in vivo*, and the tumor growth was significantly suppressed in mice immunized with this vaccine. Therefore, our findings indicate that the CS1/CRT fusion DNA vaccine may represent a promising novel myeloma therapy, and the potential for combining the CS1/CRT vaccine with other myeloma treatments.

## Introduction

Multiple myeloma (MM) is a plasma cell malignancy that accounts for 10%−15% of hematopoietic neoplasms, and 20% of deaths due to hematological malignancies. During the past several years, novel drugs (e.g., proteasome inhibitors and immunomodulatory drugs [IMiDs]) together with hematopoietic stem cell transplantation have been extensively applied in a clinical setting (1). As a result, the myeloma treatment response rates continue to improve and the survival time has been prolonged for myeloma patients. However, since myeloma remains an incurable disease, the patient will eventually relapse and die because the vast majority of the remaining tumor cells are multidrug-resistant plasma cell clones. Thus, novel treatment strategies for myeloma are urgently required. For example, aggressive multidrug combination chemotherapy, which aims at generating a complete response, strives for much longer survival and even a potential cure. However, due to the presence of treatment-related toxicity and side-effects, the improved response rates are not necessarily associated with a survival benefit. Therefore, additional attention is required to obtain a balance between treatment efficacy and patient quality of life. Therefore, novel less aggressive and more effective therapeutic approaches may represent a promising treatment direction for myeloma.

Immunotherapy is an encouraging option in the development of treatment strategies for MM, because it has a mechanism of action that is distinct from cytotoxic chemotherapy (2–6). It is important for immunotherapeutic approaches to induce a specific anti-myeloma immune response, specifically eliminate myeloma cells, and provide long-lasting protection. DNA vaccines have been demonstrated to generate long-term gene expression and induce both humoral and cellular immune responses against the encoded cancer antigens. In a DNA vaccine, tumor antigens can be presented by DNA in a suitable molecular form to elicit effective T cell-mediated anti-tumour responses. Therefore, DNA vaccines have emerged as an attractive immunotherapeutic approach for the treatment of myeloma (7–9). In addition, DNA vaccines are more economical compared to other vaccines and can be designed to encode other antigens (e.g., various immunomodulatory molecules) to enhance the resulting immune response. The safety of DNA vaccines has also been substantiated in both animal models and human clinical trials (10–12).

Although DNA vaccines are associated with several advantages, the most important factor limiting their effectiveness against cancer is poor immunogenicity. Therefore, a key property of a DNA vaccine is to select specific tumor antigens and an effective immune adjuvant which can amplify the specific anti-tumor immune response. In previous studies, the cell-surface glycoprotein, CS1 (CD2 subset 1, CRACC, SLAMF7, CD319, or 19A24), is universally and highly expressed in normal plasma cells and myeloma cells (13, 14). Plasma cell malignancy in the bone marrow, tissue, and blood all appeared to express high levels of CS1. Moreover, CS1 is not expressed in normal tissue parenchyma or in a variety of solid tumors (13). Together, these findings make CS1 an optimal target antigen for vaccination strategies against myeloma.

In this study, we also selected calreticulin (CRT, a multifunctional protein predominantly located in endoplasmic reticulum) as an immune adjuvant to amplify the specific anti-myeloma immune response elicited by the CS1-DNA vaccine. Previous studies have demonstrated that CRT plays an important role for the destruction of cancer cells *via* immune activation, and CRT exposure increases cancer immunogenicity (15–17). CRT expression on the cell surface is considered as an activating signal for multiple human cancers, whereas CRT suppression by siRNA could inhibit anthracycline-induced phagocytosis by dendritic cells and destroy the immunogenicity of tumor cells in mice.

In the present study, we constructed a DNA fusion gene vaccine (CS1/CRT) designed to target the specific myeloma antigen, CS1. We aimed to explore whether a CS1/CRT fusion DNA vaccine could induce a specific anti-myeloma immune response and control myeloma cell growth in a human plasmacytoma model.

## Materials and Methods

### DNA Vaccine Construction

The cDNA of the human CS1 gene (1,019 bp) and CRT gene (1,265 bp) were synthesized by Takara. The CS1 gene was amplified using PCR, and Hind III/EcoR I enzyme cutting sites were added to both ends of the CS1 gene. The amplified DNA fragment was cloned into the Hind III/EcoR I sites of pCDNA3.1 to generate PcDNA3.1-CS1. The CRT gene was amplified using PCR, and EcoR I enzyme cutting sites were added to both ends of the DNA fragment. Finally, the amplified DNA fragment containing the CRT gene was cloned into PcDNA3.1-CS1 to construct the DNA vaccine, PcDNA3.1-CS1/CRT, which encoded CRT linked to the specific myeloma antigen, CS1. Primer sequences for specific gene amplification are listed in **Table 1**. The accuracy of all constructs was confirmed by DNA sequencing.

**Table 1:** List of primer sequences.

Gene name	Sequence (5’ to 3’)
CS1	F: AGGGAGACCCAAGCTTATGGCTGGTTCCCCAACATG
	R: GATATCTGCAGAATTCCAAGATAACATTCTCATAGGC
CRT	F: TGTTATCTTGGAATTCTGGATGCTGCTATCCGTGCCGC
	R: TGATGGATATCTGCACACCAGCTCGTCCTTGGCCTGG

### Western Blot Analysis, Fluorescence Microscopy, and Flow Cytometry

PcDNA3.1, PcDNA3.1-CS1 and PcDNA3.1-CS1/CRT were transfected into 293T cells using Lipofectamine 2000 (Invitrogen). Following transfection for 48 h, the cells were lysed and the expression of CS1/CRT was detected by Western blot. The transfected 293T cells were respectively stained with an anti-calreticulin mAb (Abcam, ab2907) and anti-CS1 mAb (Santa Cruz biotechnology, sc-47748), observed under a fluorescence microscope (OLYMPUS IX71), and analyzed by flow cytometry (BD Accuri C6, FlowJo was used for the data analysis).

### Establishment of a Human Plasmacytoma Model

The human MM cell line, OPM2 [ATCC, with high expression of CS1 (18)] was cultured in RPMI 1640 supplemented with 10% fetal bovine serum (FBS, Gibco) at 37°C, in an atmosphere containing 5% CO_2_. OPM2 cells were collected during the logarithmic growth period. A total of 1 × 10 (7) OPM2 cells/mouse were subcutaneously injected into the right leg of BALB/c mice (Male, 5-week-old, weight 16 g−18 g, purchased from Shanghai Sippe-Bk Lab Animal Co., Ltd.). The mass growth of BALB/c mice was observed after an injection of OPM2 cells, and the tumor size was measured every other day.

### Mouse Vaccination and Tumor Challenge

A small mass was palpable under the skin of the right leg 10 days after OPM2 cells were subcutaneously injected into BALB/c mice. BALB/c mice were intramuscularly vaccinated around the mass with 100 µg DNA in 100 µL saline on day 11. The tumor sizes were measured with vernier calipers every other day, and the tumor volume (mm^3^) was calculated using the following formula: 0.5 × length (mm) × width (mm) (2). BALB/c mice were divided into three groups (n=6: 1) Group 1 was vaccinated with the pcDNA3.1-CS1 plasmid; 2) Group 2 was vaccinated with the pcDNA3.1-CS1/CRT plasmid; 3) Group 3 was the control group, which received injections of pcDNA3.1. A booster injection with the same dose was administered seven days after the first injection. Mice were sacrificed when there was the first mouse with maximum diameter of tumor up to 15 mm occurred in control group.

### Analysis of the T Lymphocyte Subsets

The splenocyte suspension was prepared after the mice were sacrificed. The percentage of CD4^+^ and CD8^+^ T cells in the splenocytes from the three groups of mice (described above) was detected by flow cytometry. The CD4^+^ and CD8^+^ T cells were also sorted by flow cytometry for the subsequent experiments which detected the CTL response against OPM2 cells.

### IFN-γ Assay

The isolated splenocytes (described above) were cultured in the upper chamber of the Transwell culture system, whereas OPM2 cells were seeded into the lower chamber. The ratio of splenocytes to OPM2 cells was 5:1. The cells were incubated at 37°C for 72 h. The supernatants were collected, and the level of IFN-γ was measured using a commercially available ELISA kit (Elabscience Biotechnology Co., Ltd, China).

### Cytotoxicity Assay *via* the Lactate Dehydrogenase (LDH)-Releasing Method

The cytotoxic lymphocyte (CTL) response against the target OPM2 cells was detected using a standard LDH method according to the manufacturer’s protocol (Cloud-clone Corp, USA). CD4^-^CD8^+^ T cells were sorted by flow cytometry (described above) and plated into 96-well U-bottom plates as the effector cells. Both the effector and target cells (OPM2 cells) were added to a final volume of 100 μl. In the experimental wells, the effector cells were co-cultured with the target cells at a ratio of 40:1 and incubated for 4 h at 37°C. Target spontaneous and maximal releasing wells were distinguished by the presence of either 100 μl medium or 2.5% Triton, respectively. The supernatant was harvested and transferred to a fresh plate to test the LDH-releasing rate. Finally, the absorbance was measured at 450 nm. The level of CTL cytotoxicity (% killing) was calculated using the following formula: A450 nm (experimental) − A450 nm (target spontaneous)/A450 nm (target maximal releases) - A450 nm (target spontaneous) × 100%.

### Statistical Analysis

An unpaired Student’s *t-*test was used to compare the data from various experimental groups. P-values < 0.05 were considered to be statistically significant.

## Results

### Identification of the Recombinant Plasmids

The DNA sequencing results showed that the human CS1 and CRT gene fragments were successfully inserted into the pCDNA3.1 plasmid to construct PcDNA3.1-CS1 and PcDNA3.1-CS1/CRT. The DNA sequencing directions and primers of inserted into the CS1 and CRT gene fragment are shown in **Supplementary Figure 1**.

### Detection of CS1/CRT Expression by Western Blot, Fluorescence Microscopy, and Flow Cytometry

**Figure 1:**
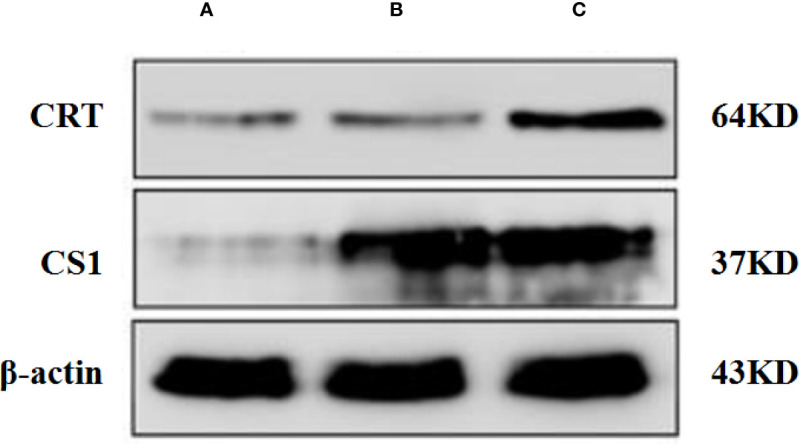
Western blot analysis. **(A)** Control, 293T cells transfected with PcDNA3.1; **(B)** 293T cells transfected with PcDNA3.1-CS1, high level CS1 expression was showed; **(C)** 293T cells transfected with PcDNA3.1-CS1/CRT, High levels of both CS1 and CRT protein expression were detected.

A Western blot was performed to detect the level of CS1/CRT protein expression in plasmid-transfected 293T cells (**Figure 1**). The Western blot results showed a high level CS1 expression in 293T cells transfected with PcDNA3.1-CS1. High levels of both CS1 and CRT protein expression were detected in 293T cells transfected with PcDNA3.1-CS1/CRT.

**Figure 2:**
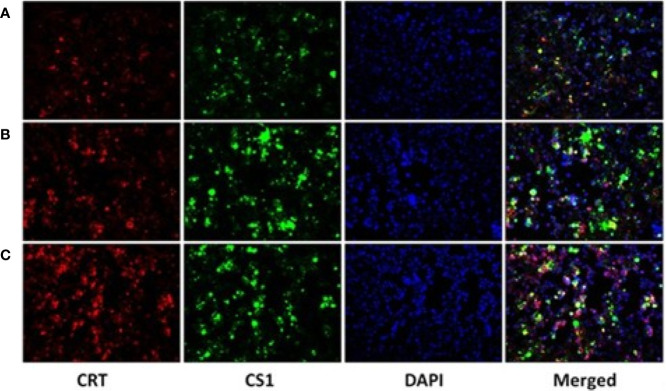
CS1 and CRT protein expression observed by fluorescence microscopy (× 200). **(A)** Control, 293T cells transfected with PcDNA3.1; **(B)** the density of green fluorescence increased significantly in 293T cells transfected with PcDNA3.1-CS1, revealing high levels of CS1 protein expression in PcDNA3.1-CS1 transfected cells; **(C)** the density of both green and red fluorescence increased significantly in 293T cells transfected with PcDNA3.1-CS1/CRT, revealing high expression of both the CS1 and CRT proteins in PcDNA3.1-CS1/CRT-transfected cells.

After the plasmids were transfected into the 293T cells for 48 h, 293T cells were collected and detected by fluorescence microscopy (**Figure 2**) and flow cytometry (**Supplementary Figure 2**). The results showed that CS1 protein was significantly expressed in the cells transfected with PcDNA3.1-CS1, whereas both CS1 and CRT protein were significantly expressed in the cells transfected with PcDNA3.1-CS1/CRT.

### Vaccination With the CS1/CRT Fusion DNA Vaccine Significantly Suppressed the Growth of Myeloma Cells

To detect the therapeutic efficacy of the CS1/CRT fusion DNA vaccine, we generated a xenograft mouse model of human plasmacytoma. Ten days after the OPM2 cells were subcutaneously injected into BALB/c mice, a small mass was palpable under the skin of the right leg in some mice. These mice were intramuscularly vaccinated around the mass with 100 µg DNA in 100 µl saline on day 11. The experimental mice were divided into three groups, which were respectively vaccinated with either the pcDNA3.1-CS1 plasmid, pcDNA3.1-CS1/CRT plasmid, or the pcDNA3.1 plasmid as a control. A booster injection with the same dose was administrated seven days after the first injection. The tumor size was measured and recorded every other day. The first mouse with maximum diameter of tumor up to 15 mm occurred in the control group eight days after the booster injection. At this time, all mice were sacrificed for further experimentation.

**Figure 3:**
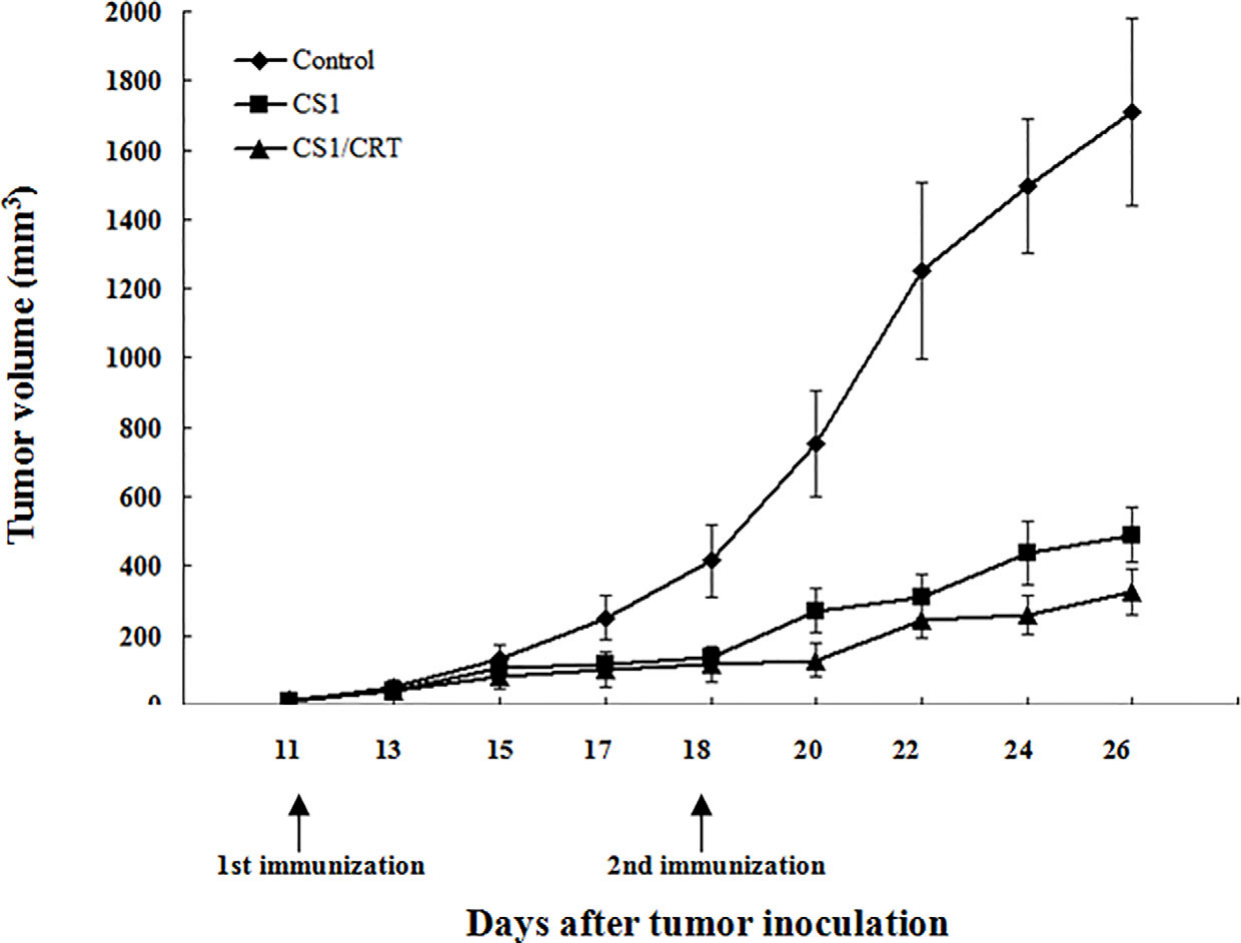
Effect of the DNA vaccine on tumor growth: The mean volume of the tumor mass in the control group (immunized with pcDNA3.1, n=6) was much larger than that of the pcDNA3.1-CS1 (n=6) and pcDNA3.1-CS1/CRT group (n=6). The group mean tumor volumes were significantly different between the two experiment groups and the control group (mean ± SD. P < 0.01, respectively), and there was also significant difference between the CS1 and CS1/CRT group (mean ± SD. P < 0.05), when the booster injection was administrated on D18 (seven days after the first injection). DNA vaccines significantly suppressed the growth of myeloma cells and the inhibition of the CS1/CRT fusion DNA vaccine on the tumor cells was more obvious than that of the CS1 vaccine.

The results showed that the mean volumes of the tumor mass in the control group were much higher than that of the pcDNA3.1-CS1 and pcDNA3.1-CS1/CRT groups (**Table 2**, **Figure 3**). These data demonstrate that the DNA vaccines significantly suppressed the growth of myeloma cells. The inhibition mediated by the CS1/CRT fusion DNA vaccine on the tumor cells was greater than that of the pcDNA3.1-CS1 plasmid, suggesting that the use of CRT as an immune adjuvant may enhance the inhibitory effect of the CS1-DNA vaccine on myeloma cells.

**Table 2:** The mean volume (mm^3^) ^1^of tumor mass following immunization.

	Control group^2^	pcDNA3.1-CS1	pcDNA3.1-CS1/CRT
D11^3^	10.34 ± 4.87	10.77 ± 3.93	11.31 ± 5.62
D13	43.43 ± 20.09	41.95 ± 16.48	43.07 ± 11.08
D15	130.17 ± 44.57	108.61 ± 30.16	81.36 ± 35.36
D17	251.30 ± 62.53	114.76 ± 26.39	100.85 ± 49.32
D18^4^	415.17 ± 104.57	138.59 ± 29.70	116.05 ± 47.66
D20	751.30 ± 152.53	271.35 ± 65.11	129.17 ± 47.09
D22	1251.30 ± 252.53	312.19 ± 62.03	242.75 ± 47.84
D24	1496.70 ± 194.04	437.01 ± 92.89	260.95 ± 54.88
D26	1707.86 ± 269.95	491.09 ± 78.02	324.96 ± 64.55

^1^Data are presented as the mean ± SD; n=6/group.

^2^BALB/c mice were vaccinated with the control plasmid, pcDNA3.1.

^3^There was no significantly different between the two experiment groups and the control group (mean ± SD. P > 0.05, respectively) in the size of tumor at the time of the first immunization.

^4^The group mean tumor volumes were significantly different between the two experiment groups and the control group (mean ± SD. P < 0.01, respectively), and there was also significant difference between the CS1 and CS1/CRT group (mean ± SD. P < 0.05), when the booster injection was administrated on D18 (seven days after the first injection).

### Analysis of T Lymphocyte Subsets

**Figure 4:**
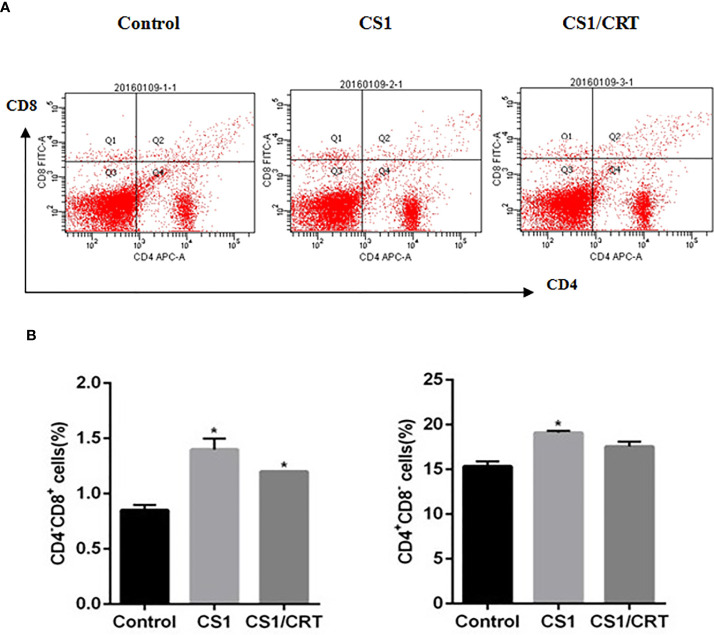
Analysis of T lymphocyte subsets from vaccinated mouse spleens. **(A)** FACS analysis was used to measure the percentage of CD4^+^ and CD8^+^ T cells. **(B)** The data are expressed as the mean ± SEM; *P < 0.05, compared with the control group.

The splenocyte suspension was prepared after all the mice had been sacrificed. The percentage of CD4^+^ and CD8^+^ T cells among the splenocytes was detected by flow cytometry. The results (**Figure 4**) showed that the percentage of CD4^-^CD8^+^ cells significantly increased in the CS1 DNA vaccine group and CS1/CRT fusion DNA vaccine group, compared with the control group (*P* < 0.05). But, there was no significant statistical difference when we compared CS1 group and CS1/CRT group (*P* > 0.05). The percentage of CD4^+^CD8^-^ cells also increased in both DNA vaccine groups, but only the CS1 DNA vaccine group was significantly different when compared with the control group (*P* < 0.05).

### IFN-γ Assay

**Figure 5:**
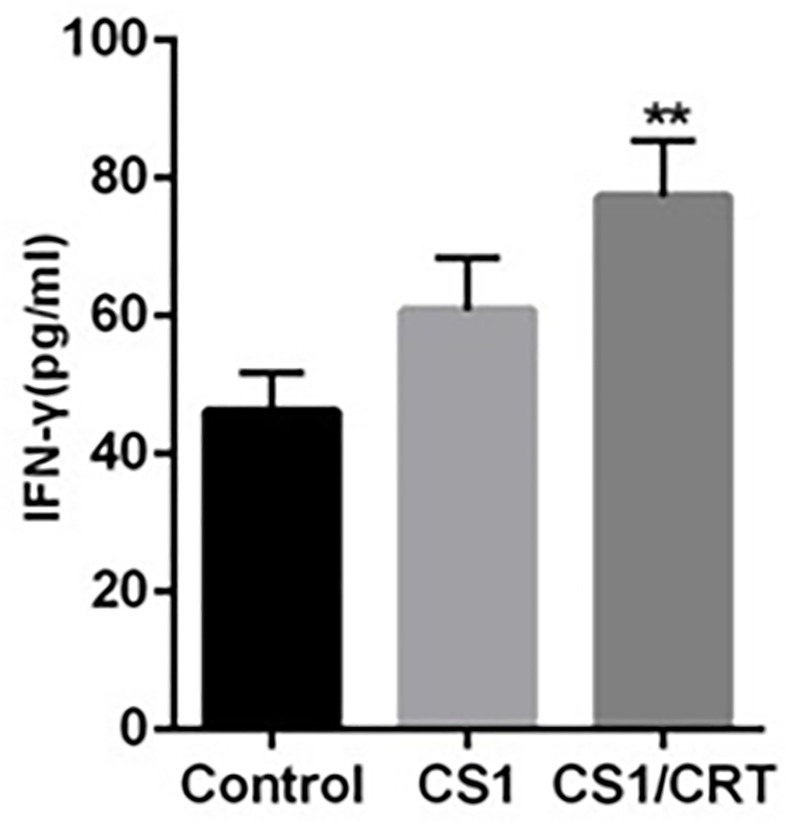
IFN-γ assay. The level of IFN-γ was using an ELISA. The data are presented as the mean ± SEM; **P < 0.01 vs control.

The results of the ELISA (**Figure 5**) revealed that the levels of IFN-γ in both the CS1 DNA vaccine group and CS1/CRT fusion DNA vaccine group were significantly increased; however, only the CS1/CRT fusion DNA vaccine group was significantly different compared with the control group (*P* < 0.01).

### Effect of the CS1/CRT-DNA Vaccine on the CTL Response

**Figure 6:**
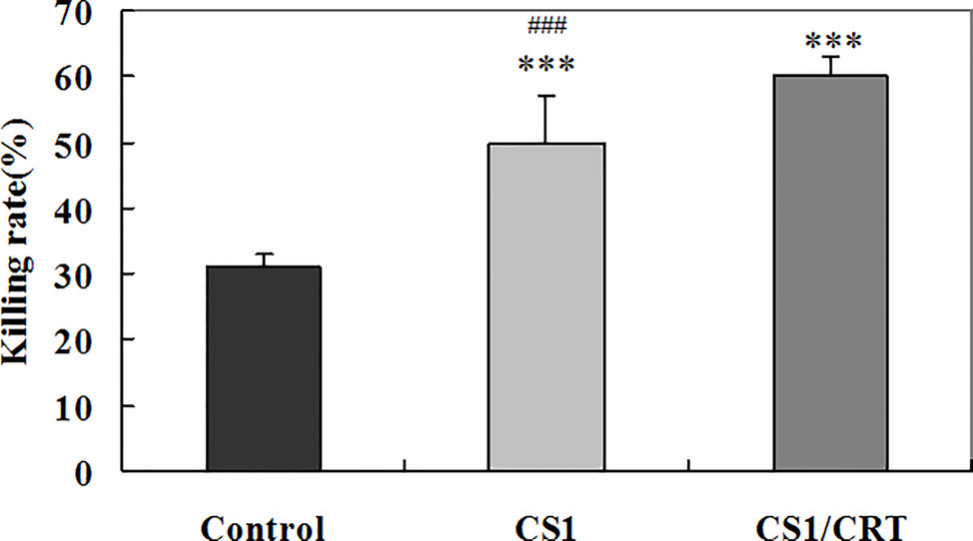
Specific anti-myeloma CTL cytotoxicity induced by the CS1 and CS1/CRT fusion DNA vaccines. The data is presented as the mean ± SD; ***P < 0.001 vs control; ^###^ *P* < 0.001 vs CS1/CRT.

To examine the CTL response induced by the CS1/CRT-DNA vaccine, we used the the standard LDH method with OPM2 cells used as target cells to detect the cytotoxicity of CD4^-^CD8^+^ cells sorted from the splenocytes by flow cytometry. The results (**Figure 6**) showed a significant difference in the killing rate of both group, compared with the control group (*P* < 0.001). These data confirm that both the CS1 DNA vaccine and CS1/CRT fusion DNA vaccine can induce a specific CTL response targeting myeloma cells. Moreover, the CS1/CRT fusion DNA vaccine induced a significantly stronger CTL response compared to the CS1 DNA vaccine (*P* < 0.001). These findings suggest that the use of CRT as an immune adjuvant can amplify the anti-myeloma immune response induced by the CS1-DNA vaccine.

## Discussion

Since MM typically affects the elderly, many patients may be too frail to undergo intensive chemotherapy (19, 20). To enhance the therapeutic effect and avoid serious complications, a combination of low intensity therapies with different mechanisms may be considered. For example, low intensity chemotherapy combined with immunotherapy may be beneficial. In general, the immune function of patients with myeloma is considered to be severely compromised, which results in an increased infection rate and reduced immune surveillance for tumor cells (21–24). The mechanisms of immune evasion of myeloma cells include the weak expression of tumor antigens, enhanced expression of inhibitory ligands (e.g., PD-L1), as well as increased numbers of regulatory T cells (Tregs) and myeloid-derived suppressor cells (MDSC), which can inhibit CTL function. Therefore, immunotherapy research for myeloma should focus on how to enhance the immune system, to recover and enhance the level of immune surveillance for myeloma cells.

Myeloma vaccines are classified as active immunotherapies, which target tumor-associated antigens and induce antitumor immune responses. Immunotherapy approaches with patient-specific protocols mean more expense, more difficult to operate, and need to take longer time. Thus, an off-the-shelf-based immunotherapy that is more cost-effective for patients is highly desired. It makes a DNA vaccine an attractive choice. The key to improve the efficacy of a DNA vaccine is associated with enhancing its immunogenicity, and promoting the T cell-mediated antitumor immune response. In our study, CS1 was selected as the specific target antigen. CS1 is highly expressed in over 95% of cases of MM (13, 18) and CS1 expression has also been found to remain high following treatment with bortezomib, or in patients who relapse after transplantation. Thus, high levels of CS1 expression are generally a universal and persistent feature in MM (14). This feature makes CS1 an attractive target for the treatment of MM. To enhance the immune response, we constructed a recombinant vector encoding CS1 and the immune adjuvant, CRT, to investigate its antitumor effects in a MM mouse model. CRT is a key determinant of the immunogenic forms of cell death, can influence antigen presentation to CTLs and promote cellular phagocytic uptake. Tumor protection requires cell surface CRT, as well as CD8+ and CD4+ T cells (15–17). Therefore, we attempted to increase CRT expression on the surface of tumor cells using a CS1/CRT DNA vaccine, thereby enhancing the T cell-mediated anti-myeloma immune response.

In our study, we constructed a recombinant plasmid PcDNA3.1-CS1/CRT. The results from the Western blot analysis demonstrated that the recombinant plasmid PcDNA3.1-CS1/CRT was highly expressed in 293T cells (**Figure 1**). Observations using fluorescence microscopy also revealed high levels of CS1 and CRT protein expression in 293T cells transfected with PcDNA3.1-CS1/CRT (**Figure 2**). Furthermore, flow cytometry revealed that the level of CS1 and CRT protein expression was significantly increased on the surface of transfected 293T cells (**Supplementary Figure 2**). Collectively, the above experimental results all demonstrate that the recombinant plasmid PcDNA3.1-CS1/CRT could transfect cells to express high levels of both CS1 and CRT protein.

In our study, to investigate the immune attack efficacy of the CS1/CRT vaccine on myeloma cells in a short term, 5-week-old male BALB/c mice were challenged with OPM2 cells (the human MM cell line, which express high levels of CS1) to establish a human plasmacytoma xenograft mouse model. The animal experiment results showed that the tumor growth was significantly suppressed in the immunized mice (P < 0.01, compared with the control group, **Figure 3**), and such suppression was more obvious in the CS1/CRT vaccine group compared to the CS1 vaccine group (P < 0.05, **Figure 3**). These findings suggest that the CS1 vaccine can effectively suppress myeloma cells, and its antitumor effects can be further enhanced by combining it with CRT as an immune adjuvant. Immunological studies revealed that an increased number of CD8+ cells among the splenocytes isolated from immunized mice (**Figure 4**). But there was no significant statistical difference when we compared CS1 group and CS1/CRT group (*P* > 0.05). The experimental data also showed markedly increased levels of IFN-γ after the splenocytes from immunized mice were inoculated with myeloma cells for 72 h (**Figure 5**). In addition, the cytotoxicity assay confirmed that our DNA vaccine can induce a specific CTL response targeting myeloma cells, and the use of CRT as an immune adjuvant can further amplify the anti-myeloma immune response induced by the CS1-DNA vaccine (**Figure 6**). So, we can see that the CS1/CRT fusion DNA vaccine induced increased production of IFN-γ and stronger CTL response, but no increase of the amount of CD8+ cells, compared to the CS1 DNA vaccine. We think that the difference may be owing to the following reasons: CRT mainly influences antigen presentation and promote cellular phagocytic uptake, while it cannot significantly increase the amount of T cells; the statistical difference may appear after the number of samples increases more.

It has historically been considered that the anti-tumor effect of immunotherapy for myeloma may be limited by the compromised immune function of myeloma patients. Some recent studies have shown that the immune system of myeloma patients with long-term disease control can recover, even to similar levels of age-matched controls (25–28). These results suggest that the immune status of myeloma patients can recover toward normal following successful treatment. This evidence provides a theoretical basis for the application of a myeloma vaccine as maintenance therapy in patients following intensive therapy to generate an effective anti-myeloma immune response and maintain long-term tumor control. Based on our results, we also consider that a myeloma vaccine may be applied as a form of pre-emptive treatment for smoldering myeloma to delay or prevent its progression into symptomatic myeloma, or for high-risk MGUS (Monoclonal gammopathy of undetermined significance) patients to prevent its conversion to MM. In addition, a myeloma vaccine can be used repeatedly to sustain an effective immune response.

Recently, some studies have shown that IMiDs combined with a cancer vaccine can enhance the anti-myeloma immune response. This effect may be due to the ability of IMiDs to enhance the immunologic milieu in patients with myeloma by promoting T cell proliferation and suppressing inhibitory factors (29–31). These results suggest that the combination of myeloma vaccines with other therapies (e.g., IMiDs) may represent a novel strategy for the treatment of refractory myeloma. Since the expression of death signals on the surface of myeloma cells induced by chemotherapeutic drugs can promote immune recognition of tumor cells, our CS1/CRT fusion vaccine combined with low dose chemotherapeutic drugs may achieve a superior anti-myeloma immune response and ultimately better tumor control (32).

In conclusion, our study demonstrates for the first time, that CS1 can be used as a target antigen in a DNA vaccine to successfully induce specific cytotoxic T cell responses against myeloma cells and suppress tumor growth *in vivo*. Furthermore, the CS1/CRT fusion DNA vaccine could enhance the anti-myeloma immune response and substantially suppress tumor growth. These findings highlight the need to explore the combination of this myeloma DNA vaccine with IMiDs or chemotherapeutic drugs for the treatment of myeloma in future studies. Thus, this study presents convincing evidence to support the application of a CS1/CRT fusion DNA vaccine in myeloma, and the potential for its use in combination with other treatments for myeloma.

## Data Availability Statement

The raw data supporting the conclusions of this article will be made available by the authors to any qualified researcher.

## Ethics Statement

The animal study was reviewed and approved by Experimental animal welfare ethics committee of Sir Run Run Shaw Hospital, Zhejiang University School of Medicine, China.

## Author Contributions

XY and WL conceived and designed the study. XY and WL performed the experiments. XY did the final statistical analysis, interpreted the data and wrote the manuscript. XY and WL obtained funding. JH contributed to the implementation of the study, scheduling of the study participants. LZ and YZ provided technical assistance. All authors contributed to the article and approved the submitted version.

## Funding

This work was supported by the National Natural Science Foundation of China (No. 81201870, 81472065).

## Conflict of Interest

The authors declare that the research was conducted in the absence of any commercial or financial relationships that could be construed as a potential conflict of interest.
