# Supplementary material for: Cytotoxic T Cell Responses Induced by CS1/CRT Fusion DNA Vaccine in a Human Plasmacytoma Model
Source: Front Oncol. 2020 Nov 20;10:587237. doi: 10.3389/fonc.2020.587237 (PMC7714938; doi:10.3389/fonc.2020.587237)
Supplement: Supplementary file 1 [file Table_1.doc]

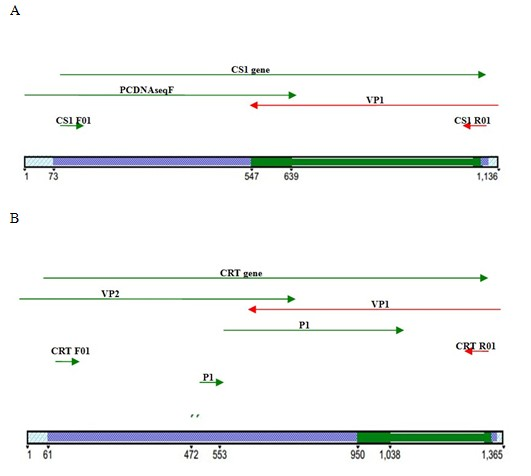


**Supplementary, Figure 1.** The DNA sequencing directions and primers of the inserted CS1 and CRT gene fragments. A: The DNA sequencing direction of the inserted CS1 gene fragment; the DNA sequencing primers of CS1 gene fragment include: pCDNA seq F 5’-AACGGGACTTTCCAAAATGTC-3’, VP1 5’-ACTTGTGGCCGTTTACGTCG- 3’; the DNA fragment encoding CS1 was amplified using a set of primers for CS1 F01 and CS1R01 (described in Table 1). B: The DNA sequencing direction of the inserted CRT gene fragment; the DNA sequencing primers of the CRT gene fragment included VP2 5’-TACGACACAATCCCTCACAC-3’, VP1 5’-ACTTGTGGCCGTTTACGTCG-3’, and P1 5’-GTTTGGTCCCGACATCTGTG-3’; the DNA fragment encoding CRT was amplified using a set of primers for CRT F01 and CRTR01 (described in Table 1).


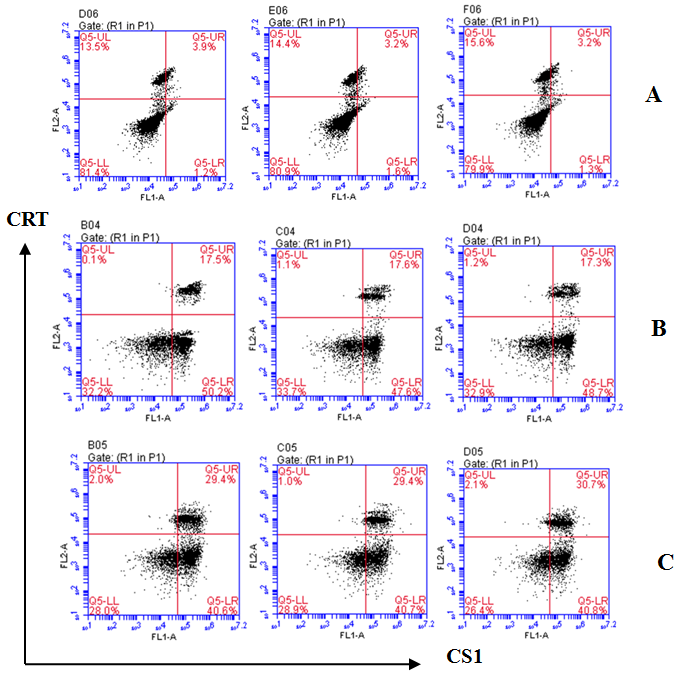


**Supplementary, Figure 2.** CS1 and CRT protein expression detected by flow cytometry. A: Control, 293T cells transfected with PcDNA3.1; B: the percentage of 293T cells transfected with PcDNA3.1-CS1, expressing the CS1 protein increased significantly; C: the percentage of 293T cells transfected with PcDNA3.1-CS1/CRT which expressed both the CS1 and CRT proteins increased significantly.
